# The Effect of Kangaroo Mother Care After Duodenal Obstruction in Neonates

**DOI:** 10.3389/fsurg.2022.813052

**Published:** 2022-05-17

**Authors:** Li-Bo Zhu, Yan-Hua Xu, Jin-Fen Li, Xue Hu, Chun-Yan Lu, Rui-Lan Li, Cai-Ping Shi, Mei Yuan

**Affiliations:** Department of Neonatology, Kunming Children's Hospital, Kunming, China

**Keywords:** kangaroo mother care, neonate, duodenal obstruction, post-operation, total enteral nutrition, length of hospital stay

## Abstract

**Objective:**

The present study aimed to explore the effectiveness of clinical application of kangaroo mother care (KMC) in neonates after surgery for duodenal obstruction in achieving total enteral nutrition (TEN) and shortening the length of hospital stay.

**Methods:**

A prospective study of 60 cases of surgery for duodenal obstruction in pediatric patients in the neonatal intensive care unit of Kunming Children's Hospital between January 2018 and December 2019 was conducted. The study subjects included 15 cases with intestinal malrotation, 18 cases with circular pancreas, 10 cases with a duodenal septum, and 17 cases with duodenal atresia or duodenal stenosis. According to the single and double numbers of the operation date, the subjects were randomly divided into the control group and observation group, with 30 cases in each group. The conventional care of enhanced recovery after surgery (ERAS) was carried out in the control group, and KMC based on ERAS conventional care was implemented in the observation group. The difference in the duration to achieve TEN and the length of hospital stay between the two groups of patients after care was compared and analyzed.

**Results:**

The average duration to achieve TEN for neonates with duodenal obstruction in the control group was 14.23 ± 3.17 days, while that in the observation group was 12.27 ± 1.15 days. The average length of hospital stay in the control group was 17.22 ± 4.71 days, while that in the observation group was 13.34 ± 2.70 days. There was a significant difference in the duration to achieve TEN and the average length of hospital stay between the two groups (*P* < 0.05). The duration to achieve TEN and the length of hospital stay in pediatric patients were significantly shorter in the observation group than in the control group.

**Conclusion:**

Kangaroo mother care has important clinical significance and application value in shortening the duration to achieve TEN and the length of hospital stay in neonates after surgery for duodenal obstruction.

## Introduction

Neonatal duodenal obstruction is one of the common gastrointestinal malformations in neonates. There is one case in 5,000–10,000 surviving neonates, accounting for around 40% of the cases of congenital gastrointestinal obstruction in neonates. Duodenal obstruction found by prenatal ultrasonography was reported at as early as 19 weeks of pregnancy in our country and it was reported at 20 weeks in other countries (1, 2). Regarding the current medical development, surgery is the only effective treatment for duodenal obstruction (3). However, surgery can cause postoperative pain, feeding difficulties, vomiting, and long-term bed rest, which are not conducive to postoperative bowel recovery. Early enteral nutrition can stimulate the recovery of the intestinal function, shorten the duration of parenteral nutrition, and reduce the incidence of complications. Studies have reported that kangaroo mother care (KMC) can improve the milk intake and immune status of children (4). KMC, also known as Skin-to-Skin Contact (SSC), is a type of kangaroo-style care for neonates that originated in Bogotá, Colombia, South America, in the early 1980s. KMC may allow the mother/father to participate in the medical decision-making and care of the child. In this type of care, the neonate's head is upright and naturally slightly flexed, and the neonate's skin is close to the mother/father's chest to increase the sense of security in neonates (5–15). KMC can allow the pediatric patients to spend the entire postoperative recovery period in the embrace and care of their parents and grow up happily, promote the growth and development of the children, and enhance the immune function and digestive function (16). This study aimed to explore the effectiveness of KMC nursing in realizing total enteral nutrition (TEN), and to evaluate whether it could shorten the length of hospital stay and achieve the goal of quality care after surgery for duodenal obstruction in neonates.

## Materials and Methods

### Sample and Data Collection

#### Sample

A total of 60 neonates with duodenal obstruction who underwent surgery for duodenal obstruction and were without severe complications in the neonatal intensive care unit (NICU) of Kunming Children's Hospital between January 2018 and December 2019 were enrolled in this prospective study. The study was explained to the parents before the initiation and informed consent was obtained from them. The present study was reviewed and approved by the Medical Ethics Committee of the hospital.

The inclusion criteria were as follows: (1) pediatric patients with a definite diagnosis of neonatal duodenal obstruction who underwent surgery without serious postoperative complications such as short bowel syndrome, wound fistula, shock, or severe infection; (2) pediatric patients with an average age of birth within 7.15 ± 10.25 days; (3) Infants with a gestational age >37 weeks and a birth weight >2,500 g; (4) pediatric patients with a 1 min Apgar score at birth higher than seven points; (5) the parents of the pediatric patient agreed to accept KMC and signed a consent form and could insist on going to the hospital for KMC every day during the hospitalization with a good understanding ability and without any mental or infectious diseases; (6) pediatric patients without congenital genetic metabolic diseases and serious diseases of the heart, kidney, liver, and other organs, such as severe infection, shock, or multi-organ dysfunction.

The exclusion criteria were as follows: (1) pediatric patients with severe infections and complicated with other factors during the recovery period that required another operation; and (2) pediatric patients with a request to give up KMC due to the reasons of the mother/father or family.

#### Data Collection

According to the single and double numbers of the operation date, the subjects were randomly divided into the control group and observation group, with 30 cases in each group. The concept of enhanced recovery after surgery (ERAS) for perioperative care was adopted in all subjects in the present study (17). The responsible nurses collected data of preoperative clinical characteristics, diagnoses, surgical methods, and postoperative fundamental state of the pediatric patients including postoperative days, whether be performed KMC nursing, and whether achieve TEN daily. In addition, length of hospital stay was calculated in the end.

### Postoperative Nursing Care

#### The Control Group

Implementation of the conventional postoperative ERAS care:

- When returning to the ward after surgery, the identification of the patient, skin integrity, wound conditions, lack of obstruction and fixation of various drainage tubes, lack of obstruction of the venous route, and consciousness after anesthesia were checked by the staff of the operating room.
- Care after anesthesia. The vital signs were observed closely, and the airway was kept open. Stability of the vital signs was maintained, and the blood gas was monitored to ascertain whether the child's internal environment was balanced and stable. Furthermore, due to the immature body temperature regulation center in the neonate, the body temperature regulation was unstable, and it was easily affected by the environmental factors. Therefore, attention should be paid to the assessment and management of body temperature.
- Care of the wound. The surgical incision was covered with a dressing (gauze), the wound dressing was kept clean, dry and fixed, and whether the wound was bleeding or exuding was observed closely. If a large amount of bright red exudation is found in the wound, the doctor should be notified in time and perform the treatment in time. The abdominal wounds were bandaged with abdominal bands, but the bandage should not be too tight to avoid affecting the breathing.
- Antibiotics were administered to prevent (control) infection.
- The effective fixation and smoothness of various pipelines was observed, and the drainage volume and color was recorded.
- The supply of nutrients was ensured, and the parenteral nutrition and enteral nutrition programs were formulated. A central venous catheter was inserted to ensure the supply of intravenous nutrition. Parenteral nutrition is continuously given by intravenous infusion to pediatric patients until enteral nutrition meeting physiological requirements and enteral nutrition is given 24 h after the operation.
- The infection management system for the unaccompanied wards was implemented, the inpatient wards were kept clean and tidy, and windows were opened regularly for ventilation. The staff in the ERAS ward should be relatively stable, and the hand hygiene of medical workers should be strictly implemented.
- Related pain was managed well. The central nervous system of newborns has matured at birth and can perceive and respond to pain. After surgery, neonates may not only experience postoperative wound pain, but also undergo various invasive operations. Pain has a certain short-term and long-term impact on the neonates. Therefore, pain should be observed, evaluated, and reported as the fifth vital sign. It is essential to conduct good pain management. Studies have shown that KMC is one of the non-drug pain interventions.

#### The Observation Group

Kangaroo mother care was conducted in the intervention group based on ERAS conventional care, and the details were as follows:

- The timing of the implementation of KMC. On the first day after the surgery, when the vital signs were stable, and with evaluation of whether there were related postoperative complications by the doctor in charge, KMC was implemented.
- Before the child was enrolled in the group, the nurse called the parents to explain the advantages of KMC, obtain informed parental consent, and inform parents of the matters requiring attention and related preparations before entering the ward. Before entering the NICU for KMC, the father/mother of the study subject was trained by nurses with an explanation of the operation method, and the operating model was used to perform practical operations. The parents were taught to accurately determine the physiological and pathological state of the neonate. The father/mother who needed to be part of the group should skillfully operate on the practical operation model for the content of nurse training.
- Notifications and related preparations. The father/mother should take a bath before going to the hospital and ensure that the skin was free of ulcers and rashes, and that they were free of diarrhea and systemic infectious diseases. They should not use perfume and special lotion, remove all jewelry, trim nails, and wear cotton and sweat-absorbing clothing. They should avoid wearing burred, tight, chemical fiber clothing. The father/mother should complete drinking, eating, and toileting before entering the ward to avoid interruption of the operation.
- Before implementing KMC, the nurse on duty prepared the environment and the child and measured the vital signs of the child, maintaining the room temperature at 26–28°C and the humidity at 55–60%. They placed a screen and a recliner beside the incubator and put a large clean and disinfected towel that could cover the recliner on it to keep the area clean. The father/mother should enter the ward with happy emotion. Before performing KMC, the nurse taught the father/mother to wash their hands follow the seven-step washing method. The mother/father was in a semi-recumbent position on the recliner to obtain a comfortable inclination, and the back could be padded with a comfortable soft quilt while assisting in adjusting the posture to make it as comfortable as possible. The nurse took the child out with the child wearing only a diaper and as much of the skin of the child exposed as possible. With the head upright, the child's body was naturally slightly flexed and placed on the mother/father's chest with the skin in contact with the skin of the mother/father and the head on the mother/father's chest. The face of the child was slightly to one side, and the body was close to the skin of the mother/father's chest, and thus the child's ear could be close to the mother/father's heart, allowing the child to hear the mother/father's heartbeat. The mother/father was instructed to lightly support the child's buttocks and back with the palm and find the most suitable and comfortable position for the child. Then, the back of the child was covered with a large towel or cotton quilt for heat preservation. All types of pipelines were fixed, and a screen was placed around the family. It was be ensured that all types of monitoring equipment were in a normal condition and could be observed by nurses. A mirror was provided to the mother/father so that the child's complexion and fingertips could be observed. During the process, the mother/father could touch the child's back and speak to the child softly.
- KMC should take 20 min the first time. During this process, the nurse would assist, evaluate, and guide the mother/father on KMC. Subsequently, the duration of KMC should be extended appropriately, with 2 h once a day.
- The mother/father participated in feeding the child once a day in the KMC after the child had received the first feed. The nurse explained some feeding-related knowledge and gave guidance when the mother/father was feeding. The amount and the type of milk were fed according to the doctor's advice, once a day, seven times a week, and continued until discharge. Breastfeeding should be performed if the disease had no special requirements for the type of milk. At the end of KMC each day, the records (vital signs, volume of milk, weight in kilograms, and length of hospital stay) were completed, and the father/mother was assisted to fill out the satisfaction survey form for KMC.

### Statistical Analysis

SPSS 23.0 was adopted for the data analysis. The countable data were expressed as *n* (%) and tested by the χ^2^ test. The measurement data conforming to the normal distribution were expressed as mean ± standard deviation (*x* ± *s*), and the independent sample *t*-test was performed for comparison between groups. *P* < 0.05 was considered statistically significant.

## Results

### Diagnoses and Surgical Methods

In the control group, 8, 10, 5, 7 pediatric patients were diagnosed with intestinal malrotation, circular pancreas, duodenal septum, and duodenal atresia or duodenal stenosis, respectively. In the observation group, 7, 8, 5, 10 pediatric patients were diagnosed with intestinal malrotation, circular pancreas, duodenal septum, and duodenal atresia or duodenal stenosis, respectively. Regarding the surgical methods, 10, 10, 4, 3, 3 pediatric patients were performed sutured anastomosis of duodenum, necrotic bowel resection and anastomosis, simple septicectomy, enterolysis, and volvulus reduction. In the observation group, 13, 8, 2, 2, 2 pediatric patients were performed sutured anastomosis of duodenum, necrotic bowel resection and anastomosis, simple septicectomy, enterolysis, and volvulus reduction.

### Pre-operative Clinical Characteristics

Pre-operative data of the two groups were collected and analyzed. No significant differences were observed in gestational age (*P* = 0.502), body weight at birth (*P* = 0.641), Apgar score at birth (*P* = 0.511), age (*P* = 0.529) and weight at surgery (*P* = 0.642) between the two groups (Table 1).

**Table 1:** Comparison of general information between the two groups.

**Group**	***n***	**Gestational age (week)**	**Body weight at birth (kg)**	**Apgar score at birth**	**Weight at surgery (kg)**	**Age (day)**
Control group	30	38.83 ± 1.01	3.31 ± 0.24	8.22 ± 0.29	3.53 ± 0.31	7.14 ± 8.44
Study group	30	38.82 ± 1.07	3.43 ± 0.25	8.31 ± 0.26	3.45 ± 0.36	7.16 ± 8.36
*t*		0.675	0.363	0.661	0.366	0.073
*P*-Value		0.502	0.641	0.511	0.642	0.529

### Nursing Care

All the children enrolled in the observation group and the control group were born in full term. Two groups of pediatric patients were given gastric tube injection feeding 24 h after the operation. In the second 24 h, 10–20 ml·kg^−1^·day^−1^ of 5% glucose water was fed to the pediatric patients for eight times. In the third 24 h, 10–20 ml·kg^−1^·day^−1^ deep hydrolyzed protein formula was fed to the pediatric patients for eight times. In the fourth 24 h: feeding amount is gradually increased according to the patient's gastrointestinal tolerance (intestinal peristalsis, defecation), and the feeding pattern gradually be changed to oral feeding as the feeding amount increases. The feeding rate was increased from the baseline concentration to the physiological requirement of 120 ml·kg^−1^·day^−1^. The control group received regular nursing according to ERAS, while the observation group received KMC nursing on the basis of ERAS routine care. All infants completed KMC within a predetermined time during the performance of KMC. The pediatric patients of the two groups were not weighed. There was no statistically significant difference in gender and performance of KMC by the mother/father (*P* > 0.05, Table 2).

**Table 2:** Comparison of the gender of pediatric patients and the KMC conducted by mother/father.

	**Gender of pediatric patients**		***P*-Value**	**Parents**		***P*-Value**
	**Male (*n* = 16)**	**Female (*n* = 14)**		**Mother (*n* = 18)**	**Father (*n* = 12)**	
Length of hospital stay (day)	13.06 ± 2.21	13.64 ± 3.09	0.152	12.99 ± 3.04	13.84 ± 2.85	0.126
Duration to achieve TEN after surgery (day)	11.82 ± 2.12	12.78 ± 2.92	0.072	12.06 ± 2.24	12.63 ± 2.12	0.141

*KMC, kangaroo mother care; TEN, total enteral nutrition*.

### Post-operative Recovery

In the observation group, the number of peristalsis per minute recovered to 4–6 times in pediatric patients 3 days after the surgery and the daily stool were 1–3 times. However, peristalsis occurred 1–2 times per minute in the pediatric patients of the control group 3 days after the surgery and stool happened at most once daily. Some pediatric patients need assistance in defecation. The length of hospital stay and the time to achieve TEN in the two groups were recorded and statistically analyzed (Table 3). The results showed that the length of hospital stay and the time to achieve TEN in the observation group were shorter than those in the control group (both *P* < 0.05).

**Table 3:** Comparison of the length of hospital stay and duration to achieve TEN between the two groups of pediatric patients.

	**The time to start KMC (h)**	**Length of hospital stay (day)**	**Duration to achieve TEN after surgery (day)**
Observation group (*n* = 30)	15.23 ± 5.12	13.34 ± 2.72	12.27 ± 1.15
Control group (*n* = 30)		17.22 ± 4.71	14.23 ± 3.17
*P*-Value		<0.001	<0.001

*TEN, total enteral nutrition; KMC, kangaroo mother care*.

## Discussion

The main discover of the present study was that KMC nursing can promote achieving TEN and shorten the length of hospital after surgery, and help achieving the goal of quality care.

### KMC Nursing Might Promote Achieving TEN

According to the results of our study, KMC nursing can promote achieving TEN. In neonates with congenital duodenal obstruction, the proximal intestine is passively dilated and there is intestinal hypertrophy, resulting in poor motility. Moreover, with the small and poor development of the distal intestine because of the obstruction, the recovery of gastrointestinal function after surgery is often slow and takes a long time; therefore, fasting is necessary for a long time. Critically diseased patients requiring long-term bed rest, together with the physiology and postoperative critical illnesses resulting in restricted mobility and other factors, will affect the gastrointestinal peristalsis, which is not conducive to the recovery of gastrointestinal function. In severe cases, it will cause a second intestinal obstruction, requiring the operation to be performed again. However, KMC could enable pediatric patients to perform passive activities, prevent intestinal adhesions, and enhance the recovery of gastrointestinal peristalsis. KMC might realize the early feeding of children, accelerate the recovery of gastrointestinal function, and achieve TEN to meet the children's nutritional needs. In addition, early enteral nutrition could effectively maintain the structure, function, and integrity of the intestinal mucosa, directly transport nutrients to the intestinal mucosal cells, promote the proliferation and repair of the intestinal mucosa in children undergoing surgery, and prevent a decrease in intestinal permeability. It could also ensure the barrier function of the intestines and reduce the migration of flora and the release of endotoxins, thereby reducing the occurrence of intestinal infections and reducing the complications of long-term use of nutritional solutions after surgery for neonatal duodenal obstruction.

### KMC Nursing Might Shorten the Length of Hospital Stay After Surgery

Based on our results, KMC nursing shorten the length of hospital stay after surgery. The application of KMC in full-term neonates and preterm neonates could maintain the stability of breathing, heart rate and blood oxygen saturation, improve the oxygenation of the child, maintain the stability of the cardiopulmonary function (12), and decrease the voice of crying and degree of wakefulness (18). KMC could also improve milk intake and immune status (4). Studies have shown that KMC is safe and has the benefits of stress reduction. It should be regarded as an essential part of providing the best care in the NICU (19). The results of the present study suggest that the KMC model could promote the recovery of pediatric patients, shorten the length of hospital stay, and reduce the financial burden of patients' families. Therefore, KMC nursing is very valuable for clinical application.

### KMC Nursing Might Help Achieving the Goal of Quality Care

Some pediatric patients were found to have duodenal obstruction during the prenatal examination and were admitted to the NICU with unaccompanied management mode from birth for the related treatment and preoperative examination preparation, surgery, and postoperative recovery period. This requires a long hospital stay, and therefore parents may feel guilty and are more likely to have anxiety and fear. However, KMC might allow the mother/father to participate in the care of their child, which could enhance the relationship between mother/father and child, improve the anxiety caused by mother–infant separation (5), improve the satisfaction of doctors, nurses and patients, and achieve the goal of high-quality nursing.

In the present study, clinical observation and comparison of whether KMC could shorten the duration to achieve TEN and length of hospital stay after surgery for neonatal duodenal obstruction were conducted. Through observation and comparison between the two groups, it was found that KMC could accelerate the recovery of gastrointestinal function to achieve TEN and shorten the length of hospital stay. In summary, KMC might have important clinical significance for shortening the duration of parenteral nutrition, promoting TEN, and shortening the length of hospital stay after surgery for neonatal duodenal obstruction. However, the present study was a survey with a small sample size, and therefore further research with a large sample size is required. Moreover, the observation index in the present study was relatively simple, and future studies should evaluate the prognostic effects of the nursing methods comprehensively.

## Data Availability Statement

The original contributions presented in the study are included in the article/supplementary material, further inquiries can be directed to the corresponding authors.

## Ethics Statement

The studies involving human participants were reviewed and approved by Medical Ethics Committee of Kunming Children's Hospital. Written informed consent to participate in this study was provided by the participants' legal guardian/next of kin.

## Author Contributions

L-BZ and Y-HX was involved in drafting the manuscript and revising it critically for important intellectual content. C-YL and MY made substantial contributions to the acquisition, analysis, and interpretation of data for the work. J-FL and XH participated in supervision and data interpretation. All authors read and approved the final manuscript.

## Funding

This work was supported by Kunming Health Science and Technology Personnel Training Project and Ten Hundred Thousand Project Training Plan 2016-SW (back-up)-80.

## Conflict of Interest

The authors declare that the research was conducted in the absence of any commercial or financial relationships that could be construed as a potential conflict of interest.

## Publisher's Note

All claims expressed in this article are solely those of the authors and do not necessarily represent those of their affiliated organizations, or those of the publisher, the editors and the reviewers. Any product that may be evaluated in this article, or claim that may be made by its manufacturer, is not guaranteed or endorsed by the publisher.
